# Disparities in pediatric obesity during COVID-19: The role of neighborhood social vulnerability and collective efficacy

**DOI:** 10.21203/rs.3.rs-3317809/v1

**Published:** 2023-09-21

**Authors:** Jungwon Min, Vicky Tam, Stephanie Mayne

**Affiliations:** Children’s Hospital of Philadelphia

## Abstract

**Introduction:**

Childhood obesity increased in the first year of Covid-19 with significant disparities across race, ethnicity, and socioeconomic status. Social distancing led to fewer physical activity opportunities but increased screen time and high-calorie food consumption, all co-determined by neighborhood environments. This study aimed to test the moderation effects of neighborhood socioeconomic and built environments on racial/ethnic disparities in obesity change during Covid-19.

**Methods:**

Using electronic health records from a large pediatric primary care network in 2018–2022, we cross-sectionally examined 163,042 well visits of 2–17 year-olds living in Philadelphia county in order to examine (1) the pandemic’s effect on obesity prevalence and (2) moderation by census-tract-level neighborhood socioeconomic disadvantage, crime, food and physical activity-related environments using interrupted-time-series analysis, Poisson regression, and logistic regression.

**Results:**

Weekly obesity prevalence increased by 4.9 percent points (pp) during the pandemic (Jan 2021-Aug 2022) compared to pre-pandemic (Mar 2018–Mar 2020) levels. This increase was pronounced across all age groups, racially/ethnically-minoritized groups, and insurance types (ranging from 2.0 to 6.4 pp) except the Non-Hispanic-white group. The increase in obesity among children racially/ethnically-minoritized groups was significantly larger in the neighborhoods with high social vulnerability (3.3 pp difference between high and low groups), and low collective efficacy (2.0 pp difference between high and low groups) after adjusting for age, sex, and insurance type.

**Conclusions:**

Racially/ethnically-minoritized children experienced larger obesity increases during the pandemic, especially those in socioeconomically disadvantaged neighborhoods. However, the buffering effect of community collective efficacy on the disparities underscores the importance of environments in pediatric health.

## Introduction

Obesity and Covid-19 were concurrent pandemics in 2020 and 2021.^1^ In the US, adult obesity prevalence rose by 3 percent points (pp)^,2^ as well as childhood obesity,^3^ from 1.7 pp^4^ to 3.1 pp^5^ increase during the first year of the pandemic.

The literature identified school closures, virtual learning, disrupted routines, increased stress, depression, and sedentary time in conjunction with decreased opportunities for physical activity and proper nutrition as reasons for the worsening childhood obesity.^6^ Studies warned that the short-term changes could become permanently entrenched.^3,7^ Notably, neighborhood socioeconomic and built environments can play an important role in obesity by determining access to safe and convenient places for physical activity, healthy food options, and community networks to get resources and information.^6,8^ During the Covid-19 crisis, residential neighborhoods acted as sources of stress or buffers against psychological distress during the pandemic through mechanisms including crime rate, parks, and collective efficacy^9^. In the early pandemic, urban adolescents had a more considerable decrease in physical activity than rural adolescents.^10^ Increased outdoor activity was more likely to occur by access to parks, even in densely populated areas.^11^ The pandemic worsened inequities in the social determinants of health through food insecurity, unemployment, poverty and lack of access to the internet.^12,13^ The structural racism resulted in disparate pandemic impacts on racially/ethnically-minoritized communities, including high Covid-19 incidence and mortality.

Philadelphia is a US Northeast megalopolis with high obesity prevalence, with significant regional-racial disparities.^14^ Philadelphia’s racial/ethnic disparities in obesity further widened by 3 pp in 2020.^4^ While previous studies have examined neighborhood moderation effects on racial/ethnic disparities in obesity generally,^6,15–17^ the extent to which neighborhood environments exacerbated or buffered against these disparities among children during the pandemic is unknown.

To address this knowledge gap, we examined the moderation effects of neighborhood socioeconomic and built environments on racial/ethnic disparities in obesity prevalence changes during the COVID-19 pandemic among children in a large pediatric primary care network in Philadelphia. We hypothesized that: 1) obesity prevalence had increased by the Covid-19 pandemic, 2) racially/ethnically-minoritized groups experienced higher obesity increases than non-Hispanic white (NHW) children, 3) neighborhood socioeconomic disadvantage and higher crime exacerbated racial/ethnic disparities in obesity increases while tree-cover, walkability, parks/playgrounds, healthy food retailers, and collective efficacy buffered it.

## Methods

### Study design and study sample

This cross-sectional study examined 2–17 year-old children’s well visits between March 2018 and August 2022 in a large pediatric primary care network across 31 clinics that serves approximately 300,000 patients annually in the Philadelphia metropolitan area. We included 73,225 patients (163,349 encounters) with a measured height and weight who lived in Philadelphia County, as Covid-19 disproportionately impacted children in densely populated areas.^10,11^ We excluded patients with implausible BMI by the modified z-scores in the CDC growth charts published in 2022 (0.2%).^18^ This study was deemed exempt by the Children’s Hospital of Philadelphia Institutional Review Board.

### Assessment and Measures

#### Obesity.

We determined the child BMI Z-score by age-specific and sex-specific BMI percentiles using the 2000 US CDC Growth Charts. Obesity was defined as ≥ the 95th BMI percentile.^19^

#### COVID-19 pre-pandemic vs. pandemic. We defined pre-pandemic from March 1 2018 to March 22 2020.

After Covid-19 hit, primary care visit volume suddenly dropped due to stay-at-home orders,^4,20^ and equipment and staff shortage^21^ continued until January 20, 2021 in the care network. We designated the intermittent period from March 23 2020 to January 20 2021. **We defined pandemic period from January 21, 2021 to the end of August 2022,** during which both the patient and clinic activities returned to normal.

#### Other patient characteristics.

Patient sex, race/ethnicity, age, and insurance type at each visit were collected from electronic health records. Patient race-ethnicity was classified as Hispanic, non-Hispanic black (NHB), NHW, and other, and was included as a marker for potential exposure to structural racism.

#### Neighborhood characteristics.

We geocoded patient’s home address and defined their residential census tracts as a proxy for neighborhoods. Neighborhood characteristics were selected based on existing associations with obesity, cardio metric health, and health inequities.^22–25^

A. Sociodemographic and economic characteristics: We used year-matched American Community Survey data, when available, to define below.

##### 1) The CDC’s social vulnerability index (SVI):

A ranking of a community’s resilience following public health disasters, based on 15 social factors in four domains (socioeconomic status, household composition-disability, minority status-language, and housing type-transportation; ranged 1 to 100, with higher scores indicating more vulnerability)^26^.

##### 2) Child’s opportunity index (COI) within metropolitan areas:

A ranking of neighborhood quality for child development via 29 neighborhood resources and conditions across three domains (education, health-environment, and social-economic; five categories, with higher categories reflecting more favorable opportunities).^27^

##### 3) Index of concentration at the extremes (ICE):

Comparing NHW households with the top quintile of US income vs. people of color households with the bottom quintile of US income to capture economic and racial/ethnic segregation (ranged − 1 indicating everyone from the deprived group to 1 indicating everyone from the privileged group).^28^

1. B. Part 1 crime: The Philadelphia Police Department defines part one crime as homicides, rapes, robberies, aggravated assaults, and thefts.^29^ We counted the total number of occurrences in each tract per year.
2. C. Physical activity-related built environments: The EnviroAtlas community map by the US Environmental Protection Agency (EPA) estimates the percentage of land covered by trees (from 0 to 100) including street trees, parks, urban forests, and single trees on various properties.^30–32^ EPA defined the National Walkability Index (ranging from 1 to 20; higher scores indicating more walkability) based on measures such as street intersection density, proximity to transit stops, and diversity of land uses.^33,34^ Percent of park/playground area within the tract was calculated using the data from OpenDataPhilly.^35^ We defined tree and park/playground as an area percentage of the neighborhood rather than the distance to the closest one, as families would benefit more from multiple choices during a long period of social distancing.
3. D. Food environments: The CDC identifies the modified Retail Food Environment Index (mRFEI) as the percent of healthy food retailers vs. the total food retailers.^36^ Fast-food expenditure ratio was calculated as the percentage of all fast-food restaurants expenditures vs. the total food-related expenditures using Esri Consumer Spending Data.^37^
4. E. Collective efficacy: Collective efficacy indicates neighborhood level of trust, cohesion, and the willingness to intervene for the common good among residents. High collective efficacy has positive impacts on mortality, as well as adolescent BMI, obesity risk, adult daily intake of fruits and vegetables, and psychological distress following the pandemic. ^9,38–40^ We employed the Southeastern Pennsylvania Household Health Survey’s social capital scale, using five relevant community questions (involvement in local groups and organizations, neighbors work together, community improvement, sense of belonging, and feelings of trust^41^ The Likert-scale responses were summed and scored between 1–10 points. After considering the survey sampling weight, we averaged the score to the tract level.

### Statistical analysis

We described patients’ individual and neighborhood characteristics at the encounter level. Using interrupted-time-series analysis with three events: 1) Covid-19 started (12th week of 2020), 2) primary care patient volume returned to normal (25th week of 2020) and 3) clinic equipment and staff shortage ended (3rd week of 2021), we explored the change of weekly obesity prevalence over time.

We further analyzed the average weekly obesity point prevalence in the above periods and their changes between the pandemic vs. pre-pandemic by age, race-ethnicity, and insurance type using logistic regression with marginal standardization to calculate the absolute changes (percent point differences) and 95% confidence intervals, and Poisson regression to show the changes on a relative scale (prevalence ratios).

To estimate the neighborhood moderation on obesity increases during the pandemic, we first examined the differences in neighborhood characteristics across racial/ethnic groups using chi-squared tests. Then we tested the interaction between the obesity increase during the pandemic and the level of each neighborhood characteristic using logistic regression, stratified by NHW and racially/ethnically-minoritized groups. All racial/ethnic minority categories were combined into one group because the distributions of neighborhood categories in the NHB and Hispanic groups were too skewed to examine their moderation on racial/ethnic-specific obesity increases.

We selected SVI, ICE, COI, part 1 crime, percent of tree-coverage, and collective efficacy as they significantly differed across racial/ethnic groups. Fast-food expenditure ratio, percent of park/playground area, mRFEI, and walkability were dropped for their low variations in Philadelphia County. In the multivariable model, we kept all neighborhood characteristics but dropped COI and ICE due to their high collinearity with SVI. COI raised colinearity issues with other characteristics as it includes many domains. SVI covers more socioeconomic characteristics than ICE. As we duplicated these models using two-level mixed effects model with children nested within clinics, models yielded trivial differences in effect estimates. Statistical analyses used STATA version 17 (Stata Corp, College Station, TX).

## Results

### Characteristics of the study population

We examined 163,042 encounters of 2–17-year-old children living in Philadelphia County between 2018 and 2022 (**Supplementary Table 1**). One-fifth had obesity; the largest racial/ethnic group was NHB (58%), followed by NHW (19%), and Hispanic (10%). More than half received Medicaid, and most lived within 30 minutes of their clinic (97%). All neighborhoods of Philadelphia County have high population density (mean: 0.009, SD: 0.004). Children’s neighborhoods were primarily low in COI (83%) and high in ICE (70%) and social vulnerability (mean (SD) = 70 (25) percentile). On average, children were exposed to 158 part 1 crimes per year (SD = 87), trees covered 19% of neighborhood land, and 16% had neighborhoods with high collective efficacy. The percentages of park/playground areas and health food stores were low, while walkability and the fast-food expenditure ratio to total food expenditure were high, with small variations.

### The trend in obesity prevalence pre and during Covid-19

**Figure 1:**
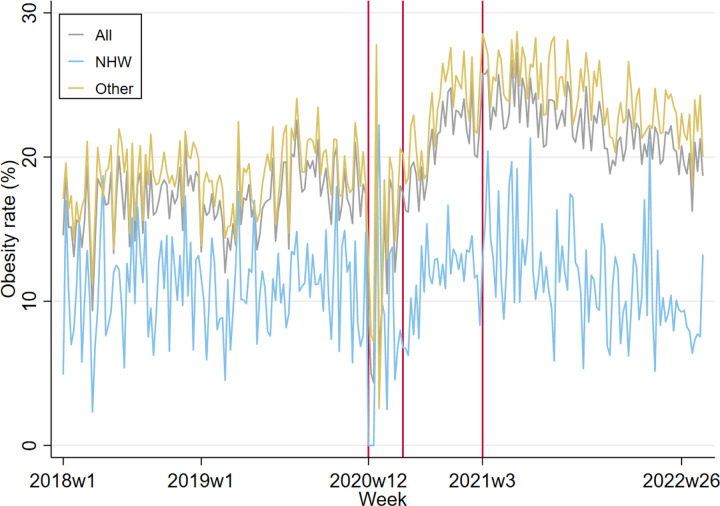
Time series line plot of obesity prevalence. Interrupted Time-Series analysis with three events: 1. Covid-19 started (3/23/2020, 12^th^ week of 2020). The intermittent period began. 2. Primary care visit volume begun to normalize (6/17/2020, 25^th^ week of 2020). 3. Equipment and staff shortage ended (1/21/2021, 3^rd^ week of 2021). The intermittent period ended and the pandemic period began.

Obesity prevalence was 17% on average pre-Covid-19 (Table 1), followed by a 9.74 pp drop when Covid-19 hit in March 2020 due to canceled primary care visits (the 1st vertical line; Fig. 1). When the primary care visit volume stabilized in June 2020 (the 2nd vertical line), obesity prevalence jumped (β(SE) = 0.74 (0.28), p = 0.009) and then steadily increased (β(SE) = 0.21 (0.05), p < 0.001). Around the time that the healthcare shortage ended in January 2021, obesity prevalence began decreasing (β(SE)= −0.06 (0.01), p < 0.001). On average, obesity increased by 4.9 pp (28% relative increases) in the pandemic (Jan 2021-Aug 2022) vs. pre-pandemic (Mar 2018–Mar 2020). All age groups, insurance types, and racially/ethnically-minoritized groups experienced increased obesity prevalence, ranging from 2.0 to 6.4 pp (16–32% relative increases), except for the NHW group. The 6–11 years old’s (6.4 pp), NHB’s (6.2 pp), and Medicaid groups (6.1 pp) had much higher obesity increases than their counterparts.

### Racial/ethnic differences in neighborhood characteristics

Children from racially/ethnically-minoritized-groups had significantly higher exposure to adverse neighborhood conditions compared to the NHW group (**Supplementary Table 2**): high in social vulnerability, extreme concentrations, and part 1 crime, while lower in COI, tree-covered land, and collective efficacy. The NHB group had the highest proportion in the most adverse categories of SVI (65% in high), ICE (29% in very high), COI (76% in very low), and part 1 crime (30% in 4th quartile), followed by the Hispanic group. The Hispanic group was more likely to be in the most adverse categories of tree-covered land (53% in < 10%) and collective efficacy (51% in low).

### Neighborhood moderation on racial/ethnic disparities in obesity increase during Covid-19

We examined the six neighborhood characteristics’ association with the change in obesity prevalence between pre-Covid-19 and the pandemic separately. Overall, SVI, ICE, and part 1 crime consistently had dose-response associations with obesity increase; a higher magnitude of obesity increase was shown in children from neighborhoods with high SVI, ICE, and part 1 crime after adjusting for individual age, sex, insurance type, and Covid-19 community transmission level (all p trends < 0.01; Table 2). However, the magnitude of obesity increase was smaller when tree-coverage, COI, and collective efficacy (all p trends < 0.01) were higher. As stratified by race-ethnicity, the NHW group had no neighborhood moderation effect on obesity increase during Covid-19. In contrast, SVI, ICE, COI and collective efficacy showed moderation effects on obesity increase among the racially/ethnically-minoritized group with a dose-response relationship.

**Figure 2:**
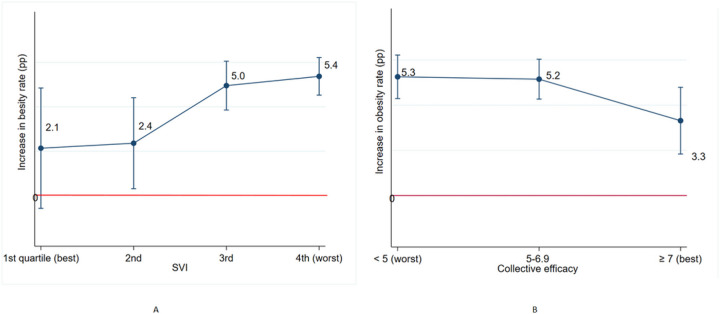
Obesity increase (percent point) during Covid-19 among racially/ethnically-minoritized children by neighborhood social vulnerability and collective efficacy in multivariate model. A. Obesity prevalence change by SVI level B. Obesity prevalence change by collective efficacy level All neighborhood characteristics in bivariate analyses (Table 2) were included, categories of SVI, part 1 crime, tree-covered land and collective efficacy, patient sex (female, male), age (2–5y, 6–11y, 12–17y), insurance type (Medicaid vs. others), and covid-19 community transmission level (high vs. others) in the multivariate model but COI and ICE were dropped due to their high collinearity with SVI. Both SVI (p trend=0.001) and collective efficacy (p trend=0.01) had a significant association with obesity increase during pandemic.

In the multivariable model, the dose-response moderation effects of SVI, tree-coverage and collective efficacy on the obesity increase during Covid-19 remained significant for the overall group (**Supplementary Table 3**). For the racially/ethnically-minoritized-group, SVI (3.3pp difference between high and low categories) and collective efficacy (2pp difference between low and high categories) remained significant moderators of the obesity increase during Covid-19, after adjusting for all other individual and neighborhood characteristics. Notably, minorities who lived in the low SVI neighborhoods did not have an obesity increase like the NHW group did (Fig. 2).

## Discussion

In this study, we found that pediatric obesity prevalence had a 5 pp increase during the pandemic (Jan 2021-Aug 2022). This increase was especially pronounced among racially/ethnically-minoritized group. Children from racially/ethnically-minoritized-groups lived in neighborhoods with greater socioeconomical disadvantages, high crime, lower tree-coverage, and lower collective efficacy on average compared to the NHW group; their higher increases in obesity during the pandemic were exacerbated by the level of neighborhood social vulnerability and buffered by high collective efficacy, even after adjusting for individual and neighborhood characteristics.

Our findings are consistent with studies from the early pandemic, which reported increased pediatric obesity and further widened racial/ethnic disparities.^4,5^. However, previous studies did not examine the impact of neighborhood environments on the racial/ethnic disparities in obesity increase during the pandemic, despite well-documented impacts of structural racism on both neighborhood conditions and the impact of the pandemic on racial/ethnic minority communities.^12,13^ The pandemic worsened inequities in the social determinants of health. Particularly high Covid-19 incidence and its mortality rate among racially/ethnically-minoritized groups included factors like crowded housing, limited English proficiency, and a high proportion of single-parent households^12,13^ which are associated with childhood obesity via lack of space for physical activity and difficulties accessing health care and health-promoting resources around the community. We addressed this research gap by presenting the different levels of obesity increase in racially/ethnically-minoritized group during the pandemic by their neighborhood sociodemographic, economic and built environmental characteristics.

Our study examined Philadelphia, a city with high obesity prevalence and racial/ethnic disparities in health. Like previous studies,^12,13^ children from racially/ethnically minoritized-groups were more likely to live in neighborhoods with high social vulnerability, crime, low incomes, and fewer child opportunities, tree-covered land, and collective efficacy. Minorities’ higher obesity increase relative to the NHW group during the pandemic differed by the level of neighborhood disadvantage. Notably, the prevalence of obesity did not increase among children from racially/ethnically-minoritized-groups who lived in the low SVI neighborhoods, which suggests the neighborhood moderation effect on childhood obesity during the pandemic.

Although SVI, COI and ICE describe neighborhood social determinants of health with different subdomains and factors, all three showed significant associations with childhood obesity increase in this study. In particular, SVI examines crowding and multi-unit structures in housing and single-parent households. COI includes access to green space, healthy food, walkability, single-headed households, and high-skill employment. These factors are directly linked with the availability of physical activity, healthy diets, the possibility of parenting burnout during the pandemic crisis, and critical for obtaining Covid-19-related information.

The frequency of crime was strongly associated with SVI, COI and ICE in this study. Frequent crime and smaller tree-coverage/green space challenge family’s outdoor activities and are associated with children’s obesity.^6^ More green spaces were associated with smaller increases in childhood obesity risk in early pandemic.^42^ However, we did not find significant associations of crime and tree-coverage with racial/ethnic minorities’ obesity increase during pandemic. We may not have enough power to detect the effect of tree-covered land on obesity increase among racially/ethnically-minoritized children. The fear of Covid-19 transmission in crowded neighborhoods may challenge racially/ethnically-minoritized families to be active outside regardless of crime rates. Although we couldn’t examine the effect of neighborhood parks/playgrounds, and walkability due to their low variations within Philadelphia, their buffering effects on childhood obesity could be less impactful than greenspace or tree-coverage because of the temporal closure of parks/playgrounds and decreased park/playground usage in early pandemic due to Covid-19 transmission risk in public spaces.

While the disadvantaged social and physical conditions may create perpetuation of inequities in pandemic obesity, it is worth noting that collective efficacy showed a buffering effect to the obesity increase among racially/ethnically-minoritized children. Our finding of the favorable influence of collective efficacy on childhood obesity is consistent with previous studies that reported lower BMI and overweight risk among adults and adolescents, and black adults with low household income,^15,38,43^ and a higher fruit/vegetable intake among mothers of toddlers receiving Medicaid^40^ who had a high level of collective efficacy. Collective efficacy has been identified as the willingness of community members to intervene together when a problem occurs.^44^ Thus, the residents in high-efficacy neighborhoods are likely to have more social interactions that result in greater conformity (e.g., adhering more to recommended guidelines for obesity-related health behaviors),^45^ and get better infrastructure and social services.^46–48^ We speculate that the residents with high efficacy may better share information, opportunities, and advocate for a healthy lifestyle during the pandemic.

### Limitations

Our data come from a single care network in one city with a majority of non-Hispanic black, which may limit generalizability. There was low variation in neighborhood walkability, park/playground areas, and food environments, which limited our ability to examine moderation by these features. We did not have enough power to detect moderation by neighborhood characteristics for more granular racial/ethnic categories and have limited years of data for COI and collective efficacy. The dataset only included children who attended well visits during the study period and thus might underrepresent children facing the highest levels of disadvantage. Further follow-up studies are needed to examine the pandemic’s long-term effect on racial/ethnic disparities in childhood obesity.

## Conclusions

During the first three years of the Covid-19 pandemic, neighborhood social vulnerability was associated with larger increases in obesity prevalence among racially/ethnically-minoritized children. However, results suggest racial/ethnic disparities could be buffered by neighborhood collective efficacy, which underscores the importance of neighborhood social environments in pediatric health. Further research is warranted to identify how to enhance the community-level interventions in improving continuous obesity disparities among children.

## Figures and Tables

**Table 1:** Changes in weekly obesity prevalence during study periods

	Pre-pandemic, %	Intermittent, %	Pandemic, %	Pandemic – Pre-pandemic difference, percent point (95% CI)	Prevalence ratio (95% CI)	interaction p
All	17.1 (2.3)	18.4 (5.4)	22.1 (2.1)	**4.9 (4.5–5.3)**	**1.28 (1.25–1.31**)	-
By age						.005
2–5y	9.8 (2.5)	10.9 (4.1)	11.8 (2.3)	**2.0 (1.4–2.5)**	**1.20 (1.14–1.26)**	
6–11 y	20.1 (3.3)	23.3 (7.2)	26.8 (3.1)	**6.4 (5.7–7.2)**	**1.32 (1.18–1.47)**	
12–17y	23.0 (3.5)	29.3 (18.6)	29.1 (3.7)	**6.2 (5.3–7.1)**	**1.27 (1.14–1.42)**	
By race-ethnicity						<.001
NHW	11.0 (3.2)	10.5 (4.5)	11.5 (3.7)	1.0 (−0.2–1.4)	1.06 (0.99–1.13)	
NHB	19.5 (2.7)	22.0 (5.9)	25.9 (2.6)	**6.2 (5.6–6.8)**	**1.32 (1.14–1.52)**	
Hispanic	20.8 (6.2)	19.3 (9.3)	25.7 (5.6)	**5.0 (3.6–6.5)**	**1.24 (1.06–1.46)**	
Other	13.5 (3.6)	14.1 (10.1)	16.1 (3.9)	**2.5 (1.5–3.6)**	**1.19 (1.00–1.40)**	
By insurance						<.001
Medicaid	19.3 (2.8)	21.0 (7.4)	25.5 (2.8)	**6.1 (5.5–6.7)**	**1.31 (1.21–1.42)**	
Other	14.6 (2.8)	15.4 (4.4)	17.1 (2.5)	**2.4 (1.8–2.9)**	**1.16 (1.12–1.20)**	

NHB: non-Hispanic black, NHW: non-Hispanic white.

Three periods: 1) Pre-pandemic (before 12th week of 2020, a total of 115 weeks), 2) Intermittent (from 12th week of 2020 to 2nd week of 2021, a total of 43 weeks), 3) Pandemic (from 3rd week of 2021 to 26th week of 2022), a total of 84 weeks).

Percentages present crude prevalence of obesity in three periods.

Percent point differences indicate the absolute change in obesity prevalence from prepandemic to pandemic period, with 95% CIs calculated by using logistic regression and marginal standardization.

Prevalence ratios and interaction p values present the relative change in obesity prevalence in the pandemic period vs. the prepandemic period by using Poisson models.

**Table 2:** Separate regression models for the association between each neighborhood characteristic and the obesity increase during Covid-19 in NHW and racially/ethnically-minoritized groups

Pandemic - Pre-pandemic obesity difference, percent point (95% CI)			
	All	Non-Hispanic white group	Racially/ethnically-minoritized group
1. SVI percentile			
Low (best)	**1.1 (−0.2, 2.5)**	1.1 (−0.6, 2.7)	**1.8 (−0.6, 4.1)**
Moderate	**1.4 (0.3, 2.6)**	0.8 (−0.6, 2.3)	**2.4 (0.7, 4.1)**
Substantial	**4.6 (3.7, 5.5)**	1.6 (−0.2, 3.3)	**5.3 (4.2, 6.3)**
High (worst)	**5.1 (4.3, 5.8)**	1.2 (−1.3, 3.8)	**5.3 (4.5, 6.1)**
P trend	**<0.001**	0.86	**<0.001**
2. ICE			
Very high (worst)	**5.7 (4.6, 6.8)**	1.9 (−3.2, 7.0)	**5.9 (4.7, 7.0)**
High	**4.8 (4.1, 5.6)**	1.2 (−0.8, 3.2)	**5.2 (4.4, 6.0)**
Moderate	**2.5 (1.4, 3.5)**	1.2 (−0.3, 2.7)	**3.9 (2.5, 5.3)**
Low/very low (best)	**0.8 (−0.3, 1.9)**	1.1 (−0.3, 2.5)	**0.7 (−1.3, 2.7)**
p trend	**<0.001**	0.97	**<0.001**
3. COI			
Very low (worst)	**4.9 (4.2, 5.7)**	2.2 (−0.4, 4.8)	**5.2 (4.4, 5.9)**
Low	**3.9 (3.0, 4.7)**	0.5 (−0.9, 2.0)	**5.0 (3.9, 6.0)**
Moderate	**2.2 (1.1, 3.4)**	1.4 (−0.1,2.9)	**3.7 (1.9, 5.5)**
High/Very high (best)	**0.6 (−1.3, 2.4)**	1.2 (−1.0, 3.3)	**0.4 (−3.0, 3.9)**
p trend	**<0.001**	0.59	**0.01**
4. Part 1 crime			
1st quartile (best)	**3.2 (2.3, 4.1)**	1.0 (−0.3, 2.3)	4.5 (3.3, 5.6)
2nd	**3.6 (2.7, 4.5)**	0.6 (−1.2, 2.3)	4.6 (3.5, 5.7)
3rd	**4.0 (3.0, 4.9)**	1.4 (−0.8, 3.6)	4.5 (3.4, 55)
4th (worst)	**5.0 (4.1, 6.0)**	1.8 (−0.9, 4.4)	5.5 (4.4, 6.5)
p trend	**0.004**	0.81	0.35
5. Tree-covered land			
< 30% (worse)	**4.5 (3.8, 5.1)**	1.4 (0.0, 2.8)	5.0 (4.3, 5.8)
≥ 30% (better)	**3.2 (2.2, 4.1)**	0.7 (−0.6, 2.0)	4.6 (3.3, 5.8)
p trend	**0.001**	0.33	0.32
6. Collective efficacy			
Low (worst)	**5.0 (4.1, 5.8)**	2.3 (−0.1,4.5)	**5.5 (4.5, 6.4)**
Moderate	**4.3 (3.6, 5.1)**	0.9 (−0.5, 2.3)	**5.2 (4.3, 6.1)**
High (best)	**2.8 (1.7, 4.0)**	0.9 (−0.8, 2.5)	**3.5 (2.0, 4.9)**
P trend	**0.002**	0.38	**0.03**

n = 135,595. Bold indicates significant trends (p trend < 0.05) in the obesity increase across environmental categories. Bivariate Logistic regression models after adjusting for patient sex (female, male), age (2–5y, 6–11y, 12– 17y), insurance type (Medicaid vs. others), and covid-19 community transmission level (high vs. others). Fast-food expenditure ratio, park/playground, mRFEI, and walkability were dropped for their low variations in Philadelphia county.

## Abbreviations

COI: Child’s opportunity index
ICE: Index of concentration at the extremes
mRFEI: modified Retail Food Environment Index
NHB: non-Hispanic black
NHW: non-Hispanic white
SVI: social vulnerability index
